# Fabrication of Ag-modified hollow titania spheres via controlled silver diffusion in Ag–TiO_2_ core–shell nanostructures

**DOI:** 10.3762/bjnano.11.12

**Published:** 2020-01-10

**Authors:** Bartosz Bartosewicz, Malwina Liszewska, Bogusław Budner, Marta Michalska-Domańska, Krzysztof Kopczyński, Bartłomiej J Jankiewicz

**Affiliations:** 1 Institute of Optoelectronics, Military University of Technology, gen. S. Kaliskiego 2, Warsaw 00-908, Poland

**Keywords:** core–shell nanostructures, hollow spheres, silver diffusion, silver-modified titanium dioxide, titania

## Abstract

Inorganic hollow spheres find a growing number of applications in many fields, including catalysis and solar cells. Hence, a simple fabrication method with a low number of simple steps is desired, which would allow for good control over the structural features and physicochemical properties of titania hollow spheres modified with noble metal nanoparticles. A simple method employing sol–gel coating of nanoparticles with titania followed by controlled silver diffusion was developed and applied for the synthesis of Ag-modified hollow TiO_2_ spheres. The morphology of the synthesized structures and their chemical composition was investigated using SEM and X-ray photoelectron spectroscopy, respectively. The optical properties of the synthesized structures were characterized using UV–vis spectroscopy. Ag–TiO_2_ hollow nanostructures with different optical properties were prepared simply by a change of the annealing time in the last fabrication step. The synthesized nanostructures exhibit a broadband optical absorption in the UV–vis range.

## Findings

In recent years, nanometer- to micrometer-sized inorganic hollow spheres (HSs) have received increasing attention due to their potential application in a variety of areas [1–4] such as solar energy conversion or photocatalysis [2,5–7]. Among them, of great interest are TiO_2_ HSs modified with plasmonic nanoparticles (NPs), which allow for the combination of the photocatalytic properties of TiO_2_ and the optical properties of plasmonic NPs [2]. This combination has been shown to extent the photocatalytic activity of TiO_2_, which is initially limited to UV light [8], to the visible or even to the NIR range of radiation [9].

Recent examples of the fabrication of plasmonic NP-modified TiO_2_ HSs are based mainly on multistep processes including hard-templating methods [10–19]. Au-modified TiO_2_ HSs of regular shape were prepared using a multistep process in which sulfonated polystyrene spheres were used as a hard-template [10]. The obtained composite catalyst exhibits a synergistic effect between the anatase crystalline shell and the AuNPs as well as superb thermal and mechanical stability of the highly dispersed AuNPs. TiO_2_ HSs decorated with ultrasmall Ag nanocrystallites and exhibiting excellent photocatalytic properties were fabricated in a multistep process including a two-step hydrothermal treatment [11]. Ag-modified TiO_2_ HSs showing efficient photocatalysis in the visible-light range were synthesized in a multistep process through a sacrificial core technique using AgBr as the core [12]. A hard-templating method with a silica template was used in the fabrication of rattle-type HSs of Au@TiO_2_ using multistep template deposition and a surface-protected etching method [13], of TiO_2_ HSs of mixed anatase/rutile composition loaded with noble metal NPs (Au, Pt, Pd) [14], and of N-doped Ag/TiO_2_ HSs [15]. A hard-templating method with a carbon template was used in a one-pot synthesis of uniform TiO_2_–Ag hybrid HSs [16], and of Ag/AgCl modified self-doped TiO_2_ HSs [17]. Finally, polymer spheres were used as hard-templates in a multistep fabrication of tri-modified TiO_2_ HSs (Ag–C/N–TiO_2_) [18], and of silver/TiO_2_ HSs [19].

Many papers dedicated to fabrication and application of plasmonic NP-modified TiO_2_ HSs have been published, including those mentioned above. In most cases the methodologies used are either complex or allow only for a limited control of the nanostructure morphologies and the resulting properties. In addition, we are not aware of any articles reporting the possibility to control the optical properties of plasmonic NP-modified TiO_2_ HSs by means of changes in their morphologies. In this work, we present the results of studies on the fabrication of Ag-modified TiO_2_ HSs with broadband optical absorption through controlled silver diffusion in Ag–TiO_2_ core–shell nanostructures (CSNs). Our approach comprises three simple steps starting from the synthesis of the metallic core, through its coating with titania and finally annealing leading to plasmonic hollow nanostructures with plasmon resonance in a broad spectral range. SEM microscopy was used to visualize changes occurring in the morphology of the nanostructures. XPS spectroscopy was used to provide information regarding the chemical composition of Ag-modified TiO_2_ HSs.

**Figure 1:**
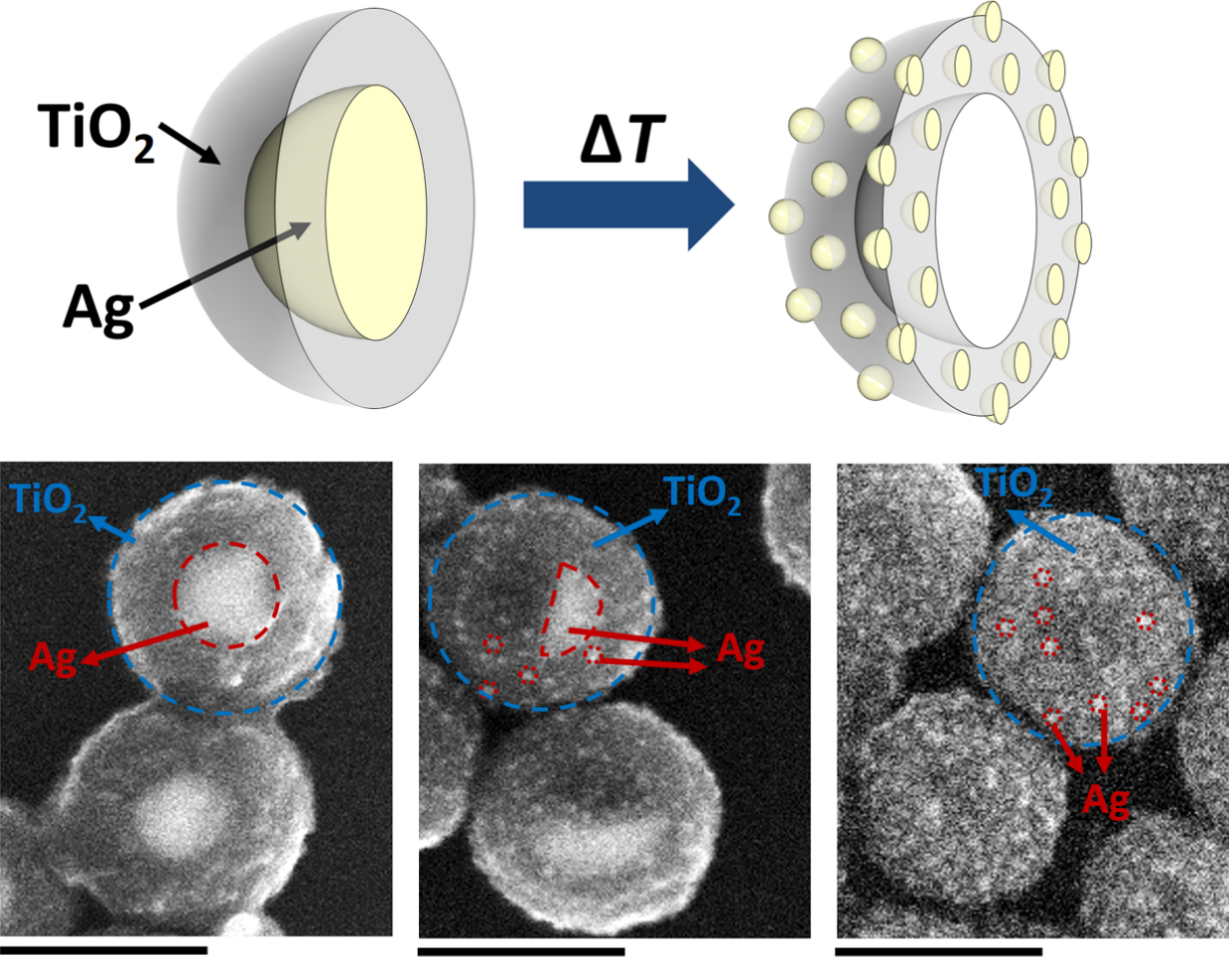
Formation of Ag-modified TiO_2_ HSs (top) and SEM images showing Ag–TiO_2_ nanostructures at different stages of their fabrication (bottom, scale bar = 200 nm).

**Figure 2:**
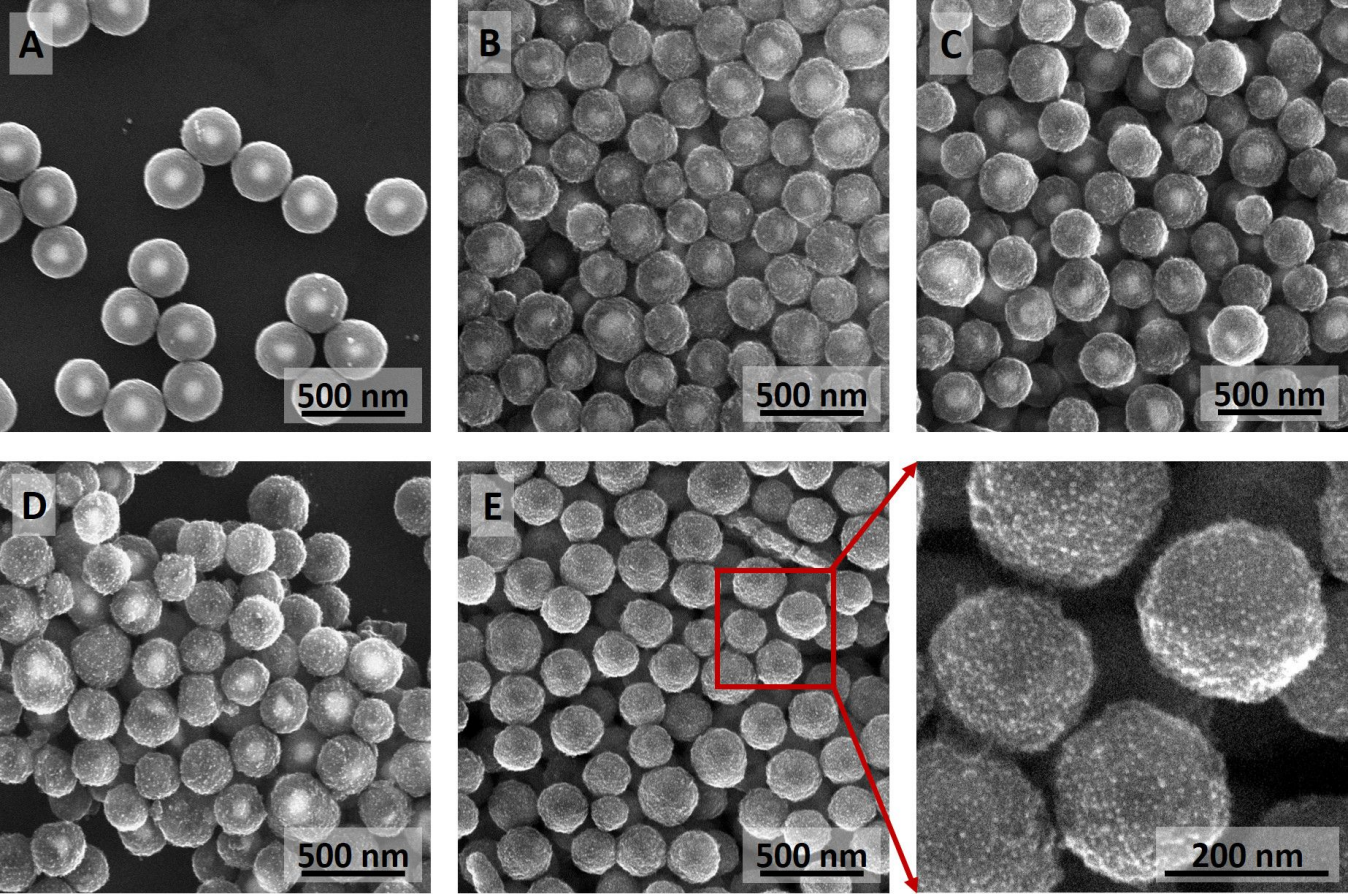
SEM images of freshly prepared Ag–TiO_2_ core–shell nanostructures (A) and Ag–TiO_2_ core–shell nanostructures after annealing at 150 °C for 0.5 h (B), 1.5 h (C), 3 h (D), and 12 h (E).

Figure 1 shows a schematic illustration of the formation of Ag-modified TiO_2_ HSs from Ag@TiO_2_ CSNs together with SEM images of the structures at different stages of their fabrication. We have demonstrated recently that AgNPs can be covered in a controlled manner with a smooth titania shell of variable thickness by the hydrolysis of organic titania precursors to form Ag@TiO_2_ CSNs [20]. Examples of such structures are shown in Figure 1 (down left) and in Figure 2A. Interestingly, further thermal modification of these fabricated Ag@TiO_2_ CSNs yields unexpected results. Annealing of Ag@TiO_2_ nanostructures in a muffle furnace results in Ag diffusion from the silver core into the titania shell. As a result, the gradual diminishment of the silver core (DP50 = 113 nm) and the formation of much smaller Ag particles (diameter below 10 nm) on the titania shell surface are observed. The metal diffusion into the titania shell has not been observed in the case of Au@TiO_2_ CSNs fabricated using the same method as Ag@TiO_2_ CSNs. This is likely due to much lower reactivity of Au compared to Ag. Ag–TiO_2_ nanostructures at an intermediate and the final stage of thermal modification are shown in SEM images in Figure 1 and Figure 2B–E.

The SEM images in Figure 1 clearly indicate the titania shell, the Ag core and products of its thermal transformation, i.e., disappearing Ag cores and newly formed Ag nanoparticles. In the first image (Figure 1, bottom left) the Ag core is clearly visible as a bright circle. Upon thermal transformation, the Ag core becomes smaller and, depending on the initial CSNs and the annealing time, it will take on different shapes (Figure 1, bottom centre). It must be stated here that transformation does not occur identically in all CSNs in the given samples, as can be seen in Figure 2, and resulting optical properties will be a sum of the properties of all particles in the sample. If CSNs are annealed for a sufficiently long time the Ag–TiO_2_ nanostructures shown in Figure 1 (bottom right) and Figure 2E are formed. These nanostructures are empty inside as indicated by a darker region of low average atomic number in the middle of the particles. They also have a high number of small AgNPs (much smaller than the size of the initial Ag core, below 10 nm) present on the surface of the titania shell.

The process of formation of Ag-modified TiO_2_ HSs can be controlled simply by the annealing time, and as a result, nanostructures of different morphologies can be obtained (Figure 2). At shorter annealing times (Figure 2B–D) the diffusion in Ag–TiO_2_ CSNs does not occur at the same speed. This is indicated by the presence of Ag–TiO_2_ nanostructures with cores at different stages of size reduction, including nanostructures with intact cores (Figure 2B–D). However, after longer annealing times in all Ag–TiO_2_ CSNs, silver cores were fully converted to Ag species (i.e., positively charged Ag clusters as discussed below or other subnanometric silver species) embedded in a titania shell or smaller AgNPs located at the shell surface (Figure 2 E).

To the best of our knowledge, the silver diffusion in TiO_2_ nanostructures has been only investigated in the case of TiO_2_ thin films [8,21–24] and our studies are probably the first to discuss this process occurring in Ag@TiO_2_ CSNs. Based on our results and the diffusion mechanism proposed for AgNPs on titania films [8,21], the silver diffusion in Ag@TiO_2_ CSNs can occur in the following manner. Titania is oxidized in an air atmosphere and as a result, holes, which may further oxidize AgNPs surrounded by the titania shell, are generated. Upon oxidation, AgNPs gain a positive electric charge and may eject positively charged Ag clusters. In case of Ag@TiO_2_ CSNs and depending on the annealing time, these ejected Ag clusters may either be accommodated within or on the titania shell. In the titania shell, these silver clusters could react with the TiO_2_ host through the formation of a complex [Ag–(TiO_2_)] [21]. Upon reaching the surface of the titania shell positively charged silver species could also form AgNPs much smaller than the core of the initial Ag@TiO_2_ CSNs. These small AgNPs are responsible for the observed optical properties of Ag-modified TiO_2_ HSs. The proposed mechanism of silver diffusion and especially the importance of oxygen molecules are supported by the fact that when freshly prepared Ag@TiO_2_ CSNs are placed in the XPS spectrometer and annealed under vacuum, without the presence of oxygen, no silver diffusion is observed.

**Figure 3:**
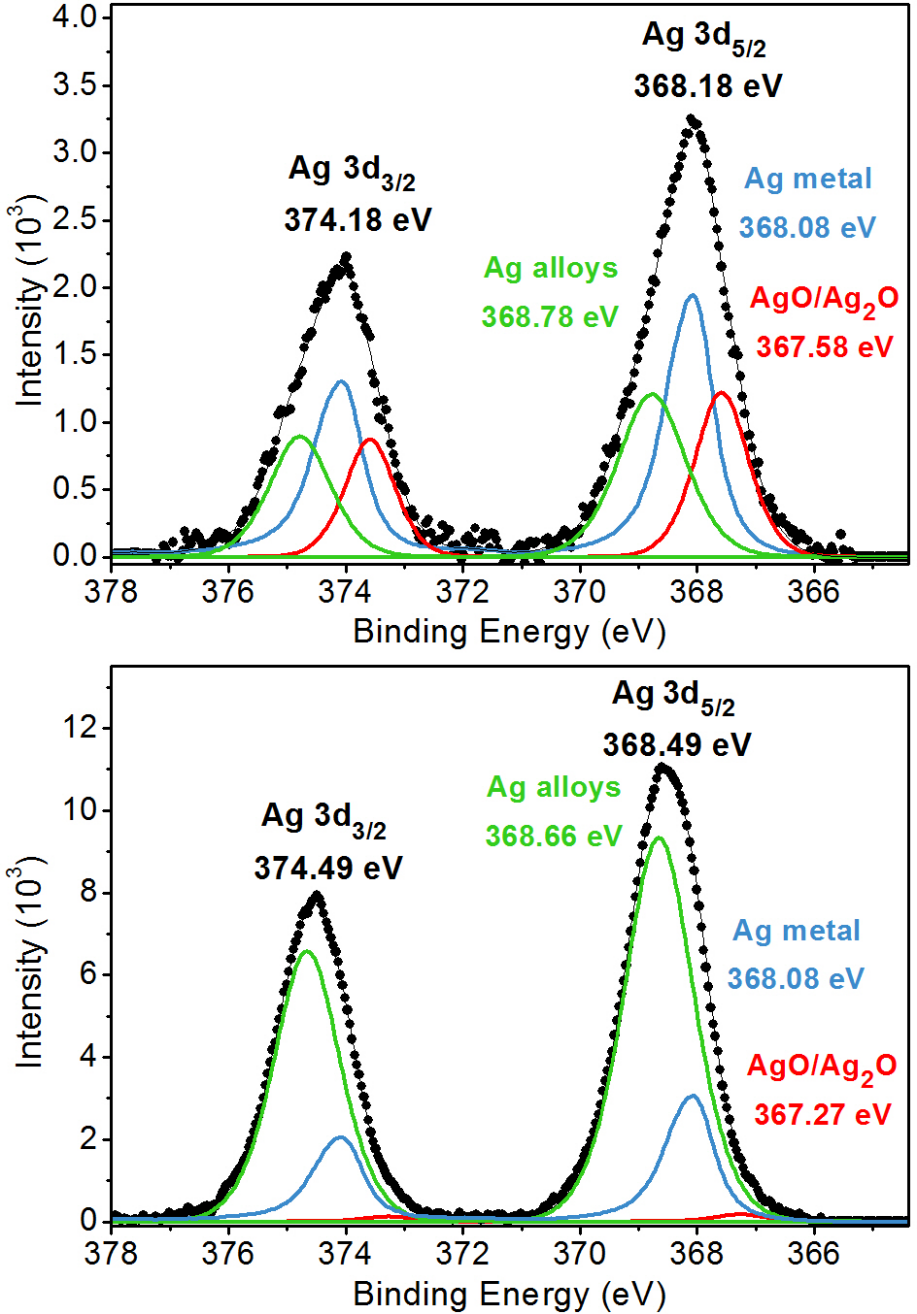
XPS Ag 3d_1/2_ and Ag 3d_5/2_ spectra of freshly prepared Ag–TiO_2_ core–shell structures (top) and after annealing at 150 °C for 12 h (bottom).

The analysis of the Ag 3d band shows that silver is present in Ag-modified TiO_2_ HSs in at least three different forms, i.e., Ag metal, oxides (AgO/Ag_2_O) and probably in the form of alloys (Figure 3). This observation suggests that silver is present in fabricated nanostructures not only in the form of AgNPs on the surface of the hollow TiO_2_ spheres, but is also embedded in the other forms within the TiO_2_ HSs. The position and shape of the peaks of metallic silver were determined based on measurements of a silver plate with a purity of 99.97%. The half-width of the Ag 3d_5/2_ peak of metallic Ag is approximately 0.95 eV and its maximum lies at 386.08 eV, which is consistent with the data in the literature [25]. The peak associated with silver oxide appears at lower binding energies than the peak of metallic silver [26–27]. Due to the very small offset for different forms of silver oxide, only one peak located between 367.27 and 367.58 eV with a half-width of 1.03–1.07 eV was modeled in the spectra. The third peak with a maximum between 358.66 and 358.78 eV and a half width of 1.36–1.40 eV was assigned to a silver alloy with titanium. Its location cannot be linked to any oxidation state of silver. Such a shift of the Ag 3d_5/2_ peak can only come from alloys of silver with metals of lower electronegativity [28]. Wang and collaborators obtained this effect for an Ag/Al alloy in which Ag and Al have electronegativity values of 1.93 and 1.61, respectively [28]. We believe that a similar situation occurs in the case of Ag–TiO_2_ nanostructures due to the electronegativity of Ti (1.54) being lower than the electronegativity of Ag.

**Figure 4:**
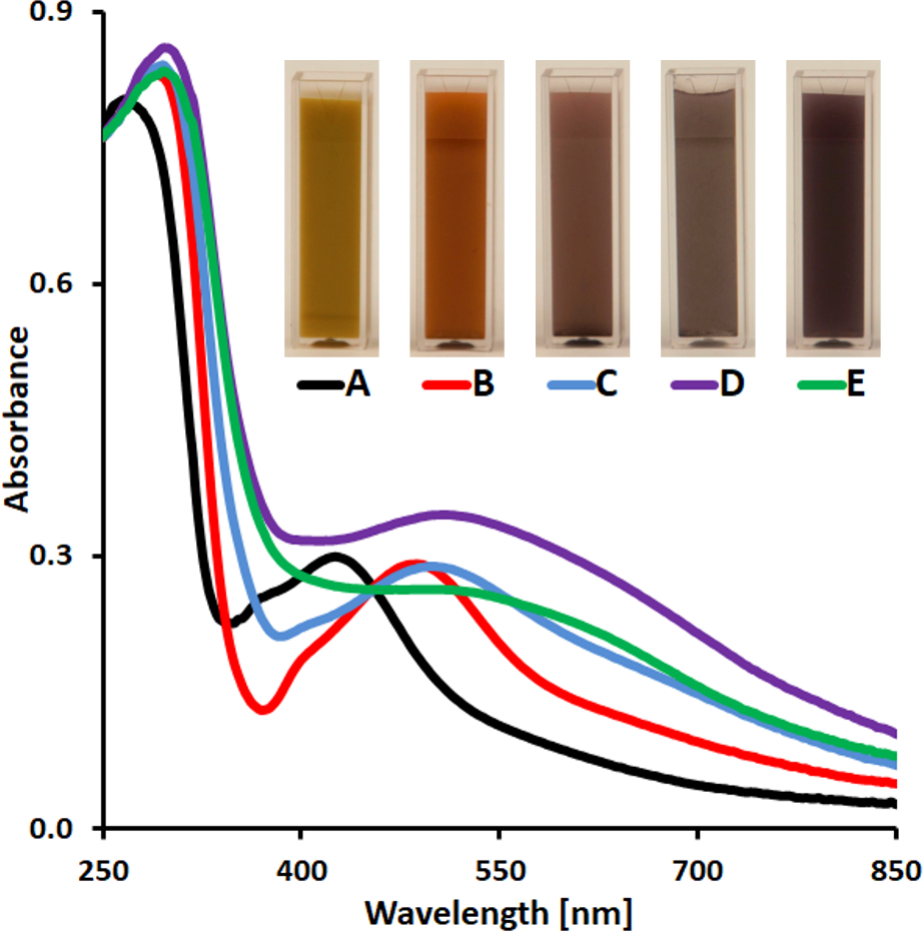
UV–vis spectra and images of aqueous suspensions of freshly prepared Ag–TiO_2_ core–shell nanostructures (A) and after annealing in 150 °C for 0.5 h (B), 1.5 h (C), 3 h (D) and 12 h (E).

The UV–vis spectra of aqueous suspensions of freshly prepared Ag@TiO_2_ CSNs (Figure 4A) and nanostructures resulting from their annealing at 150 °C for 0.5 h (Figure 4B), 1.5 h (Figure 4C), 3 h (Figure 4D) and 12 h (Figure 4E) are shown in Figure 4. To better visualize the optical properties of the fabricated CSNs and Ag-modified hollow TiO_2_ nanostructures, images of their aqueous suspensions are also shown in the inset of Figure 4. As we have shown previously, coating of AgNPs with TiO_2_ leads to an overall increase in the refractive index of their local dielectric environment and, as a result, to a red-shift of the plasmon resonance [20]. As can be seen from curve A in Figure 4, Ag@TiO_2_ CSNs have a broad absorption in the UV–vis range. This is the characteristic absorption of these composites, which does not change remarkably with a change of the shell thickness. The annealing of Ag@TiO_2_ CSNs leads to nanostructures with significantly changed optical properties as can be seen from curves B–E in Figure 4. In all cases, a red-shift of the maximum of absorption and a strengthening and widening of the plasmon resonance bands were observed. It is significant that the Ag-modified TiO_2_ hollow nanostructures show a broad and relatively strong absorption in the whole investigated spectral range, which corresponds to over 60% of the energy in the solar energy distribution [29].

The changes observed in the optical properties of the Ag-modified TiO_2_ HSs compared to Ag@TiO_2_ CSNs are related to several changes occurring in the nanostructure morphology. Any interactions affecting the optical properties of these CSNs occurred only between a single silver core and the titania shell. Based on the proposed mechanism, the annealing gradually led to a transformation of the single and relatively large core (DP50 = 113 nm) into multiple and tiny silver clusters, which with increasing annealing time were moving through the titania shell toward its edge and were finally forming AgNPs of different size. Therefore, the resulting optical properties represent the sum of the change of number, size and shape of the AgNPs, their local environment (in the final structure many small AgNPs are located on the interface between TiO_2_ and water), and the plasmon–plasmon coupling between the AgNPs on the titania shell [21].

In summary, we have shown herein a simple and template-free approach to the fabrication of Ag-modified hollow TiO_2_ spheres via controlled silver diffusion in Ag–TiO_2_ CSNs. Ag–TiO_2_ CSNs of different shell thicknesses, which are fabricated via a previously developed method, can be further modified by annealing to form Ag-modified hollow TiO_2_ spheres with different morphologies. Hollow nanostructures with a variable amount of AgNPs on the shell surface and with different optical properties resulting from the structural differences can be fabricated simply by control of the annealing time. Fabricated Ag-TiO_2_ hollow nanostructures exhibit a broader and larger optical absorption in the UV–vis range than the Ag–TiO_2_ CSNs they are made from. In addition, a significant number of AgNPs can be observed on their surface and, therefore, based on the existing literature, these nanostructures should be of great interest for applications in solar light-driven photocatalysis and photovoltaics [30].

## Supporting Information

File 1 Materials and methods.
