# Supplementary material for: Fabrication of Ag-modified hollow titania spheres via controlled silver diffusion in Ag–TiO2 core–shell nanostructures
Source: Beilstein J Nanotechnol. 2020 Jan 10;11:141–6. doi: 10.3762/bjnano.11.12 (PMC6964658; doi:10.3762/bjnano.11.12)
Supplement: File 1 — Materials and methods. [file Beilstein_J_Nanotechnol-11-141-s001.pdf]

BEILSTEIN JOURNAL OF NANOTECHNOLOGY

Supporting Information

for

Fabrication of Ag-modified hollow titania spheres via controlled silver diffusion in Ag–TiO_2 core–shell nanostructures

Bartosz Bartosewicz, Malwina Liszewska, Bogusław Budner, Marta Michalska-Domańska, Krzysztof Kopczyński and Bartłomiej J. Jankiewicz

Beilstein J. Nanotechnol. **2020,** *11,* 141–146. doi:10.3762/bjnano.11.12

Materials and methods

License and Terms: This is a supporting information file under the terms of the Creative Commons Attribution License (https://creativecommons.org/licenses/by/4.0). Please note that the reuse, redistribution and reproduction in particular requires that the authors and source are credited.
The license is subject to the *Beilstein Journal of Nanotechnology* terms and conditions: (https://www.beilstein-journals.org/bjnano)

Materials

Titanium(IV) butoxide ($C_{16}H_{36}O_4Ti$, >97%, TBT) and hydroxylamine hydrochloride ($NH_2OH·HCl$, >99%) were purchased from Sigma Aldrich. Acetonitrile (C_2H_3N, >99.5%) and ethanol (C_2H_6O, >99.8%) were obtained from Avantor Performance Materials Poland S.A. Nitric acid (HNO_3, 65% w/w aqueous solution) and hydrofluoric acid (HF, 40% aqueous solution) were purchased from Chempur. Methylamine (CH_5N, 40% w/w aqueous solution) and silver nitrate ($AgNO_3$, 99.9%) were purchased from Alfa Aesar. All chemicals were used as received without additional purification. Ultrapure deionized (DI) water (18 MΩ·cm at 25 °C, Hydrolab, Poland) was used throughout the experiments. All glassware was treated before use with titania etching solution ($HF/HNO_3/H_2O$ = 1/4/15 v/v/v) for 5 min and rinsed several times with DI water.

Synthesis of silver–titania core–shell nanostructures

The silver–titania core–shell nanostructures were synthesized using a method described elsewhere [1]. In the first step, AgNPs with DP50 (median) particle diameter equal to 113 nm were synthesized by the reduction of silver nitrate with hydroxylamine hydrochloride. Next, a small volume of the concentrated aqueous colloid solution of AgNPs was transferred into a mixture of ethanol and acetonitrile. Coating of the AgNPs was carried out by hydrolysis and condensation of titanium(IV) butoxide onto metallic cores. The particles obtained were centrifuged and washed several times with ethanol. Next, the suspension of core–shell particles was placed in a Petri dish and dried in air at 50 °C for 24 h.

Fabrication of Ag-modified hollow TiO_2 spheres

Ag-modified hollow TiO_2 spheres were fabricated via controlled silver diffusion in Ag–TiO_2 CSNs. The diffusion of Ag from the core into the titania shell was initiated by annealing freshly prepared Ag–TiO_2 CSNs. A typical experiment was performed as follows: 0.2 g of Ag–TiO_2 powder in a ceramic crucible was placed in a muffle furnace preheated to a temperature of 150 °C. Next small portions (ca. 0.05 g) of the powder were collected after time periods of 0.5, 1.5, 3 and 12 h. Before further characterization all collected samples were dispersed in 5 mL of water using an ultrasonic homogenizer. The optimal annealing temperature was determined experimentally, and it is a compromise between the time of the process and the possibility of its control. Although at higher temperatures annealing occurred faster, it was difficult to obtain nanostructures in intermediate states between core-shells and hollow spheres.

Characterization of Ag–TiO_2 nanostructures

Morphology and size of all fabricated nanostructures were examined with field-emission scanning electron microscopy (FEI Quanta 3D FEG Dual Beam) using an Everhart–Thornley detector (ETD) in custom mode at an accelerating voltage of 30 kV and a probe current of 70 pA. To prepare samples for SEM imaging, the fabricated Ag–TiO_2 nanostructures were first ultrasonically dispersed in water and then the suspension was dripped on a silicon wafer and dried in air before analysis. The chemical composition of Ag–TiO_2 nanostructures, CSNs, and hollow spheres was determined using X-ray photoelectron spectroscopy (XPS, Prevac XPS/UPS System). The measurements were carried out using an Al anode generating X-ray radiation with a photon energy of 1486.6 eV. In order to investigate the phenomenon of silver diffusion in the Ag–TiO_2 CSNs, XPS measurements were performed on the CSNs

samples prepared right after synthesis and after annealing in a muffle furnace. The samples for XPS measurements were prepared in the same way as for SEM imaging. The optical properties of fabricated Ag–TiO_2 nanostructures were determined using UV–vis spectroscopy (Perkin Elmer Lambda 650 UV–vis spectrophotometer). All measurements were carried out at room temperature in a spectral range of 250–850 nm and using a 1 cm optical path quartz cuvette. For the measurements, the cuvette was filled with a suspension of the fabricated Ag–TiO_2 nanostructures and placed inside an integration sphere. By carrying out measurements in the integration sphere we were able to determine the pure absorbance of the fabricated nanostructures.

References

1. Bartosewicz, B.; Michalska-Domańska, M.; Liszewska, M.; Zasada, D.; Jankiewicz, B. J. *Beilstein J. Nanotechnol.*, **2017**, 8, 2083–2093. doi:10.3762/bjnano.8.208
